# Finished with Life Anyway and Then Stigmatized for Attempting Suicide—An Overview

**DOI:** 10.3390/healthcare10112303

**Published:** 2022-11-17

**Authors:** Jill Julia Eilers, Erich Kasten

**Affiliations:** Department of Psychology, Faculty of Human Sciences, Medical School Hamburg, 20457 Hamburg, Germany

**Keywords:** suicide, suicidality, stigmatization, healthcare professionals

## Abstract

This article provides an overview of suicide and how society deals with it. Starting from early societal imprints through historical, religious and political influences, the origins of stigmatization are addressed. Even today, suicidal people experience stigmatization not only from society but also from the health system that treats suicidal people. This has far-reaching consequences for the people affected and runs counter to optimal treatment. Different approaches to a possibly improved handling of suicidality will be discussed.

## 1. Introduction

The reasons for suicide are manifold and have always been the subject of philosophical as well as scientific theories. Furthermore, more people die as a result of suicide than of HIV, malaria, breast cancer, war or homicide worldwide every year [1]. The purpose of this review article is to provide a historical-religious/-philosophical and -social overview of the origins of stigmatization of suicidal people. Religious views, the concept of “honour” and the question of the “meaning of life” have shaped the general stigmatization of suicide in the past and will be elaborated in this review [2].

According to the guidelines of the Center of Disease Control and Prevention (CDC), the following definition exists: “Suicide is death caused by injuring oneself with the intent to die. “Suicidality means the sum of all thought and behaviour patterns of people who strive for their own death in their thoughts, through active action or passive omission or by refraining from action, or who accept it as a possible result of their actions.” [3]. This definition is based on the assumption that suicidal tendencies can occur in principle in all people, but are usually manifested by mental disorders and psychosocial crises. This is considered as the “medical-psychosocial paradigm of suicidality” [4]. It should be emphasised that suicide is to be understood as a constriction of thinking, feeling and acting—triggered, for example, by (subjectively) experienced crises, psychological or somatic suffering or by societal and social guidelines and requirements. Suicidal acts often occur as an impulsive act within a small time span of only a few minutes after a trigger (conflict with a spouse, death of a relative, etc.). However, there is often a longer-lasting development or planning. This development is often characterised by a longer phase of ambivalence. Even in highly suicidal patients, there is often a residual ambivalence that leaves the outcome of the suicide attempt open to some extent [5]. It can make sense to make certain hotspots difficult to access. High bridges, skyscrapers and railway lines are often the first port of call for spontaneous, impulsive suicides. If the time span of acute suicidal tendencies of only a few minutes is taken into account, blocking the hotspot can lead to a subsiding of the suicidal impulse [6]. Suicide is usually preceded by the so-called presuicidal syndrome. The syndrome comprises the three characteristics of constriction, increased aggression and flight into thoughts of death, which regularly precede a suicidal act [7]. There is a special case here, the so-called rational suicide: this kind of suicide is based on a rational decision without any psychopathological background. Genuine rational suicides, however, are rarely found. In most cases, a psychopathological background (e.g., narcissistic injury, depressive disorder) can also be recognised in apparent rational suicides [8,9].

However, the definition of suicidality does not cover suicides committed in the context of tribal rituals (e.g., elderly people refusing to eat in order not to be a burden to the community), political or social motivation (e.g., kamikaze suicides of Japanese pilots in World War II) or religiosity (e.g., martyrdom) [10]. Nevertheless, politically or socially motivated suicides, as well as societies general handling of suicidality, have a considerable influence on the stigmatization of those affected and the associated suicide rate, as will be described in more detail later.

A suicide (attempt) or suicidal tendencies affect the person directly because of the psychological suffering, but also because of the social treatment of suicidal tendencies and suicidal people. There is a widespread tendency towards stigmatization, which can reinforce suicidal tendencies and can extend not only to the suicidal person themselves, but also to their relatives and friends: A commited suicide affects on average six other people [11]. The diverse social backgrounds of this will first be presented in the following as part of a historical overview, before the various backgrounds of stigmatization will be discussed. 

## 2. Historical Overview of the Origin of Stigmatization of Suicidal People

The term “stigma” comes from ancient Greek and means “sore mark”. Historically, stigma was associated with the branding of slaves, criminals or traitors and served the purpose of making them publicly identifiable so that such persons would be avoided [12]. Goffman [13] established the term “social stigma” in this context to describe the discrediting of a person or group that differs from prevailing cultural norms.

Even in the ancient world, the moral assessment of suicide was controversial and the subject of heated debates among scientists, politicians and philosophers. Socrates (469–399 BC), Plato (428–347 BC) and Aristotle (384–322 BC) rejected suicide because of religious and ethical points of view (Plato & Graeser, 2012). Seneca (1–45 AD), on the other hand, considered suicide as an appropriate option if a situation required it—and in classical Athens (480–323 BC), suicide could be authorised by the authorities [14].

The first social backgrounds without philosophical connotations can be found above all in Asia, whereby at the same time one of the major exceptions in dealing with the evaluation of suicidality can be found there: In Japan there is a special form of ritual suicide (so-called “seppuku”) with the aim of restoring honour for oneself or one’s family after “transgressions”. Although seppuko was banned by law in Japan in 1873, it continued to be practised by the population. Finally, Japanese military propaganda used the seppuko, which was associated with “honour” among the Japanese population, for its own purposes during World war II: Japanese military pilots died by “kamikaze” (“gods’ wind”) attacks, which meant attacking enemy units with no chance of survival [15]. Japanese society is generally described as having little stigma towards suicide [16]. However, research by Kawashima et al. [17] has found that a romanticised notion of suicide in the Japanese population, created by the socio-cultural background, has contributed to Japan having the highest suicide rate in the world.

From the end of the Middle Ages, access to “medical” perspectives was also found in Europe. According to this, suicidality was a “mental illness”, which, however, was and still is also regarded in a highly stigmatised way. This was evident, for example, in the first psychiatric hospitals, which primarily provided for the isolation of those affected in order to protect the general population from the mentally ill [18,19].

## 3. Religious Backgrounds and Legalisation of Suicide

Religion and laws, although the two have been linked for a long time and in some countries still are, also influence suicide-related views in the population [20]. Therefore, the view of suicide of the major world religions will now be described. In order to shed more light on the legal situation in this regard, the so-called “assisted suicide” and “passive” as well as “active euthanasia” will be considered in addition to the criminal law view of suicide. 

According to Buddha’s teachings, every person should freely dispose of his or her life. Therefore, suicide is not forbidden and is not a disgrace. If someone commits suicide because they cannot cope with their problems, these problems are carried over into the next life. In Buddhism, it is generally true that anyone who destroys life worsens their karma and thus puts obstacles in their way on the path to enlightenment. This also applies to people who commit suicide. However, Buddhists believe that they improve their karma if they can help deeply desperate people and dissuade them from their intention to take their own lives. Every Buddhist is even called upon to help in this way [21]. 

Relatively similar is the handling of suicide in Hinduism: almost everyone will be punished in the next life with illness or other bad conditions if they take their own life. However, some ascetics also end their lives themselves. They abstain from eating until they starve to death. However, they do not do this out of desperation, but for religious reasons. By renouncing, they show that they are spiritually strong and above worldly and physical things. According to Hindu belief, these ascetics do not actively take their own lives, but accept death by renouncing food. For this reason, many Hindus even worship these ascetics. People who take their own lives by starving or dying of thirst are therefore not viewed negatively. Until about 200 years ago, a special suicide even caused admiration among Hindus: Widows who had themselves burnt alive with their deceased husbands were therefore highly respected. Widow burning, also called sati, is a femicide in Hindu religious communities. This ritual was most common in India, but they also occurred in Bali and Nepal. Some of these women were held in high honour after their death and sometimes worshipped as gods. Furthermore, their family gained high prestige. [22]. Originally, the women of princely families whose husbands had died in battle killed themselves in this way, possibly to avoid falling into the hands of the enemy. In the course of time, however, widow burning was demanded in many circles of the population. Widow burning was particularly common among the Kshatriya castes, such as the Rajputs in northern India, where it still occurs today [23]. According to Indian law, any direct or indirect support of widow burning is now prohibited. Traditional glorification of such women is also punished. However, this law is not always implemented equally [24].

While there are no references to the stigmatization of suicidal people in the original Judeo-Christian writings, i.e., the Torah or the Bible [25], the condemnation of suicide by the Church finally prevailed from late antiquity onwards, which is in complete contrast to the handling of suicide for example in Buddhism [26]. The church’s approach to suicide led to excommunication and sanctions of suicidal people (even posthumous after a completed suicide) as well as their family members. Those who successfully took their own lives were buried outside Christian cemeteries [27]. The Bible assumes that life was given by God and therefore may only be ended by God. Anyone who ends it himself goes to hell as a sinner [28]. In France, Louis XIV enforced a far more aggressive punishment of suicides: The body of the deceased was dragged face down through the streets and then hung up or thrown on a rubbish heap, which was a special cruelty for the bereaved. Furthermore, all the person’s property was confiscated [29]. Since the Middle Ages, so-called “pastoral counselling” has been offered by the church within the framework of the Christian tradition [30]. According to Ziemer [30], pastoral care represents the communication between a “person seeking advice” and a “person helping”. The goal is the strengthening of the Christian faith and the turning away from sins—which includes suicidality. The positions of religious faiths towards suicidal behaviour seem to have an influence on suicide prevention as well as on the form of social stigmatization towards suicidal people. The social adherence to norms and moral values associated with higher religiosity can be seen as a protective function for suicide—and indeed empirical evidence shows that if a person is religious, this is associated with a lower risk of suicide. However, strong affinity to religiosity is also associated with higher stigmatization tendencies of suicidal actions and people [31,32,33]. 

The view of Islam is very similar to that of Christianity: According to Islam, suicide is considered a sin. For Muslims, it is certain that Allah gives life and takes life. Furthermore, the Koran emphasises that Allah helps people in difficult situations and that they can trust in him. According to Islam, the body is on loan from God, who is the actual owner of the body. Therefore, it is fundamental that people should preserve their bodies and prevent death. The time of death is therefore left to God, and a violation of this is considered a sin and will be punished in hell [34]. 

As mentioned above, the legal aspects of suicide also influence stigmatization. In most European countries suicide was still punishable until the 20th century [35]. The Netherlands became the first country in the world to legally authorise both assisted suicide and active euthanasia in 2002 [1]. Passive euthanasia is the denial of medical treatment necessary to sustain life. In 2005, passive euthanasia was legalised in France. In April 2009, Luxembourg decided that doctors who practice passive euthanasia can be prosecuted neither under criminal law nor under civil law. In Spain, Norway, Hungary, England and Sweden, passive euthanasia is possible to a limited extent [36]. Active euthanasia involves the administration of lethal substances, such as the injection of a lethal agent. However, there are cases where the distinction between passive and active euthanasia is disputed. This includes, for example, the administration of painkillers for severe pain, which have a lethal effect if the dosage is too high. Here, it is debated whether this is passive or active euthanasia [37].

Assisted suicide means ending one’s own life with the assistance of another person. The concept usually refers to suicide that is assisted by a medical doctor, the so-called physician-assisted suicide. Physician-assisted suicide is legal in some countries under certain circumstances, including Germany, Austria, Belgium, Canada, Spain, Switzerland, Luxembourg, The Netherlands, New Zealand, some parts of the United States and some parts of Australia. Switzerland has a unique status with regard to assisted suicide: Here, assisted suicide is not only permitted for local patients, but also for foreign patients. In fact, many people travel there with the aim of ending their lives, which is called suicide tourism [38]. Constitutionally, assisted suicide is legalised in Germany and Italy, as well as in Columbia, but their governments have not yet legislated its practical implementation [39,40]. 

Worldwide, these forms of euthanasia are also legally permitted in Belgium, Luxembourg, Canada and in some US states (Washington, California and others) [40]. In the BeNeLux states, in addition to euthanasia in the case of “incurable illness”, euthanasia in the case of mental disorders is also permitted, in compliance with certain legal requirements. In the period from 2011 to 2014 in the Netherlands, 66 people between the ages of 30 and 70 who were classified as “mentally ill” ended their lives through active or assisted euthanasia. In this context, 55% of the euthanasia recipients were classified as depressive, 25% had psychotic symptoms in the foreground, 42% were diagnosed with post-traumatic stress disorder or an anxiety disorder and four of the euthanasia recipients were diagnosed with “cognitive deficits” by the medical profession. The legal prerequisite of “capacity to consent” and “capacity to judge” to obtain euthanasia was assumed to be given by the attending physicians [41]. However, the existence of this precondition of a “freely” decided or responsible suicide (“rational suicide”) is disputed among psychiatrists and psychotherapists [42,43]. Furthermore, in the Netherlands active euthanasia in the case of dementia—even against the will of the person affected—is considered legal. The prerequisite for this is a living will before the onset of the illness [44]. 

In Germany and in the United States as well as in many other countries, there is a so-called “suicide clause”: this is a clause in a life insurance policy that regulates the non-payment of the sum insured. It often contains provisions regarding, e.g., a waiting period (which often lasts several years), and, in addition, deals with the suicidal person’s “state of mind”, which often can be determined only posthumously: For most insurance companies, insanity is an exclusion-criteria for the payment of the sum insured to the surviving dependants [45]. In the case of recourse to euthanasia, the suicide clause is legally controversial—this also concerns so-called “passive euthanasia”. Passive euthanasia includes the refusal of life-sustaining measures, such as artificial respiration during a serious illness. However, it can then become problematic with regard to claiming insurance benefits—especially if the relative who has to decide about the life-sustaining measures terminated is the same person as the beneficiary of the life insurance [46,47]. In this case, of “almost-suicide”, insurance benefits may be withheld. 

In summary, historical developments and finally jurisprudence show that there have been controversial views on suicidality since antiquity. Considered a sin and/or a mental illness, and punishable by law in western cultures until far into the 20th century, the stigmatising, prejudiced attitude towards suicidality is also reflected in current social views, as current studies show. After all, jurisdiction sets norms and rules and interacts with societal views. Japan’s approach to suicidality is a particular exception: Suicidality is little stigmatised there, but suicide rates are particularly high because suicide is socially suggested as an honourable solution to the social understanding of “shame” or “failure” [16,17].

## 4. Structural Stigmatization

In view of the legal situation described and the social/historical and religious backgrounds, the concept of so-called “structural stigmatization” towards suicidal people becomes exemplarily clear: Structural stigmatization comprises the negative consequences towards people who are discredited on the basis of various characteristics and attributes by political decisions and laws as well as by other public or private institutions as well as by the church [48]. Due to their social (“structural”) position, these public and private institutions have a social power that can systematically negatively affect the lives of suicidal people and their relatives and discriminate against them. Discrimination here means the devaluing and disadvantaging of a social group or individual on the basis of characteristics such as gender, skin colour, origin, illnesses or stereotypical views of society [49]. The term “structural” here describes the large social reach of, e.g., authorities, offices or also service providers in the health care system. Furthermore, structural stigmatization also establishes a social orientation for dealing with suicidal people, as has already been described. However, there are legal foundations that are supposed to prevent any kind of discrimination, which also includes structural discrimination, for example The Universal Declaration of Human Rights (UDHR) [50] and the Basic Law for example in Germany (GG) (Art. 3) [51]. In 2006, the General Equal Treatment Act (AGG) came into force in Germany and included the factors of structural and institutional discrimination for the first time [51]. Thus, it can be summarised that although a number of legal provisions have been created to mitigate and prevent structural discrimination—and as a part of this also the stigmatization of suicidal people—stigmatization can still be found in society and politics.

## 5. State of the Research and Stigmatization Concepts

According the conceptualization of to Link and Phelan [52] stigma is basically the result of a combination of five components: The first component serves to identify interpersonal differences (state of health, skin colour, etc.). Here, a social selection process takes place, according to which differences are either classified as relevant or irrelevant. First, the social relevance is assessed before stigmatization takes place: for example, high blood pressure is a socially accepted and widespread phenomenon, while people with mental diseases as, e.g., schizophrenia are considered “dangerous” and “unpredictable” and are thus strongly stigmatized [53]. The second component is stereotyping: The person in question is attributed by special characteristics that usually have negative connotations. The third component in the stigmatization process is categorization into “the others”—the stigmatized group—and “us”, the “normals”. The fourth component is discrimination and loss of status, which means that people experience devaluation, rejection and social exclusion due to stigmatization. The fifth component of the stigmatization process is the use of power. According to Link and Phelan [52], the consequences of social power would become clear if it is theoretically assumed that a stigmatized group attempted a stigmatization: For example, psychiatric patients often refer to their caregivers as “pill-pushers”—a “cold and arrogant” “the others” who must be “watched out for” “. However, the patients lack the social, economic and political power to translate their stigmatization of the professionals into consequences. Therefore, professionals can hardly be considered a stigmatized group under these circumstances [52]. In terms of stigmatization towards suicidal people, this would mean that suicidal people would be perceived as having characteristics which distinguished them from “normal” (i.e., “non-suicidal”) society. On this basis, stereotypical views of suicidal people would be formed, such as “people with suicidal thoughts have a weak will”, which would lead to negative emotional reactions towards this group (e.g., from professionals in psychiatric institutions: “I am annoyed by suicidal people”) [54]. According to the social psychological definition, stigmatization is considered to be a part of human information processing and is expressed through behavioural, cognitive and affective processes. Analogous to the concept of Link and Phelan [52], stereotypical views of the stigmatized group are formed through cognitive processes. The formation of stereotypes also serves to classify social views about the respective stigmatised groups [55]. In this way, the process of forming a comparatively time-consuming, elaborate judgement is replaced and a simplified as well as generalized image of the other person is created [56]. In the context of the process just described, negative stereotypes are also linked to negative effects and form the emotional level of stigmatization. Finally, stigmatization manifests itself at the behavioural level in the form of open discrimination against the stigmatised group, which is usually accompanied by hostile behaviour: e.g., by labelling the stigmatised group as weak in character, incompetent or even dangerous, as well as by deliberately disadvantaging them or avoiding contact [55].

## 6. Stigmatization of Suicidal People as an Effect Factor for Suicidality

Suicidal people often avoid seeking professional help, especially due to stigmatization, as they fear a socially “negative” evaluation—for example, the view of being “weak”, “cowardly”, “irresponsible” and “stupid” [57]. Here, the stigma experienced by those affected has a particularly negative impact on their health and well-being, as they are confronted with negative emotional reactions by the stigmatizing environment [58]. Thus, actually suicidal people are often also objectively and not only subjectively labelled by their environment as “weak”, “selfish” or “incapable” and are objectively ascertainable—not only subjectively perceived—ostracized by their social environment [48]. A study by Corrigan et al. [59] from the USA underlines these findings: The authors’ vignette-based online survey revealed that suicidal persons were socially evaluated as “weak” and “crazy” and, in addition, evoked the emotions of “fear” and “anger” in their fellow human beings. Furthermore, the subjective experience of stigmatization causes the internalization of the stigma content (“self-stigmatization”) in the stigmatized group. This is followed by an attribution of the negative evaluation by the social environment to one’s own person and thus a social devaluation directed against oneself. Those affected therefore often withdraw from their environment out of shame and try to keep their suicidality a secret, which hinders the use of therapeutic help and consequently further reduces the quality of life—which in turn promotes suicidal acts [48,58,59]. In line with these findings, Oexle et al. found that experiences of stigmatization can increase suicidality in people with mental illness. Survivors of suicide attempts face both the stigmatization of mental illness and the stigmatization of suicide, which may contribute to an increased risk of completed suicide. To address this, the authors interviewed 13 survivors of suicide attempts about the experiences and consequences of stigmatization. Stigmatization resulted in significant emotional distress and fostered loneliness and hopelessness, which have been identified as important factors in suicidality. The findings suggest that both the stigmatization of mental illness and the stigmatization of a suicide attempt may contribute to suicidality among people with mental illness in general and survivors of suicide attempts in particular [60].

The findings of Pitman et al. [61] are also interesting here: The bereaved of a suicide committed by a friend or family member are also more likely to become suicidal. This increased suicidality results from the avoidance of seeking psychological support due to the initially socially experienced and subsequently internalized stigmatization. Perceived stigmatization is generally considered to be one of the main factors for suicide among people suffering from a mental disorder—in this case, suicide is seen by those affected as the only way to escape stigmatization from environment. Especially compared to people with schizophrenia, social stigma is seen as a highly relevant trigger for suicide [62,63,64]—in particular, affective disorders and disorders with psychotic symptoms show the highest suicide rates among mental disorders, whereby according to the WHO [65], the risk of suicide is highest in schizophrenia [63]. Thus, it is not the mental disorder per se but the stigmatization by the social environment that is the decisive factor leading to suicidality in those affected [62,64,66]. The results of Breheny’s [67] study exemplify societal stigmatizing attitudes and associated social discrimination towards mental disorders: The study compared the willingness of a group of healthy participants to interact with people suffering from either schizophrenia, depression or skin cancer. Participants showed a greater willingness to interact with skin cancer patients, while people diagnosed with schizophrenia or depression were avoided. With regard to suicidal people and social exclusion, a study conducted in Germany by Ludwig et al. [68] found that the local population is more likely to be hostile to suicidal people in their family or close circle of acquaintances than to suicidal people from their wider circle of acquaintances (e.g., colleagues, co-workers, neighbours). Schomerus et al. [69] confirmed the connection between stigmatization towards people with mental disorders and suicide as a result of stigmatization. Their study found that age-standardised suicide rates were negatively correlated with social acceptance (β = −0.46, *p* = 0.014), implying that low social acceptance of mental disorders is associated with higher suicide rates. The comparison of social acceptance of people with mental disorders with suicide rates as well as socioeconomic status of people with mental disorders from 25 European countries also provides evidence that stigma contributes greatly to explaining the variance in suicide risk and rates across countries. 

## 7. Stigmatization as a Stressor

According to the authors Schomerus et al. [69], social stigma acts as a stressor and cause of social isolation for people with mental disorders, which promotes suicidal behaviour. Accordingly, those affected frequently experience both, a lack of support and a lack of understanding from their closer social environment, which results in an increased social withdrawal. The associated isolation and hopelessness as well as the anticipation of negative events ultimately contribute to suicidality as social stressors [70]. Additional evidence regarding the relationship between stigma and suicidal behaviour is provided by Lehmann, Hilimire, Yang, Link & DeVylder [71]: In a large-scale study, the authors used interviews and self-reports to examine suicidality, the perceived stigma of people with mental disorders, secrecy about mental disorders, and the perceived hopelessness of those affected. Many of the participants had already been referred to psychiatric institutions in the past, where a mental disorder had been diagnosed. In this context, the social stigmatization of people with mental disorders had contributed to the suicidal tendencies of those affected. As a possible explanation for this, the authors cite the connection between perceived stigmatization and one’s own secrecy about the mental disorder—whereby secrecy out of fear of social stigmatization in particular seems to bring negative emotional consequences with it. This can be considered a fundamental social stressor.

## 8. Suicidality as a Symptom in Mental Disorders

**Figure 1:**
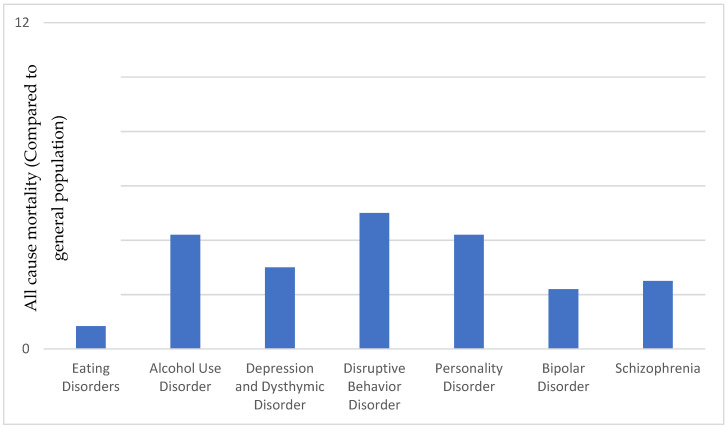
Mental Disorder.

The numbers collected on the extent to which suicidality and mental illness are connected vary considerably, since on the one hand a suspected diagnosis is often only made after the suicide has been committed and only the suicide and retrospective information from interviews with relatives are used for the diagnosis. This increases the risk of incorrect information. On the other hand, some studies only consider deceased suicidal patients with already known mental disorders, which means that a strong selection of the available data has already taken place in advance [72]. According to the WHO [65], about half of all suicide-related deaths in Western cultures are caused by mental disorders. Thus, affective disorders—primarily depression—are present in the medical history of about 50% of all completed suicides. According to the ICD-10, suicidality is explicitly part of the diagnostic criteria for depression, which exemplifies the close link between suicidality and affective disorders [73]. On the other hand, this criterion-based diagnostic interlocking already shows that suicidal thoughts and/or suicidal actions are decisive factors for the need for psychotherapeutic treatment. Chesney et al. [74] conducted an analysis of 407 reviews with 1.7 million patient records. They found that all mental disorders are associated with increased suicide risk. The highest risk was found in anorexia nervosa patients and addiction patients, followed by depression, bipolar and borderline (Figure 1). Furthermore, people with somatoform disorders are just as likely to commit suicide as people with depression, which is often underestimated [75]. Somatoform disorders are physical complaints that are not due to an organic disease and are assumed to be caused by the soul, for example by high mental tension, stressful experiences or interpersonal conflicts [73]. Medical and psychological psychotherapeutic professionals are involved in the medical treatment of affected patients. Studies show that almost half of later suicidal persons were still undergoing general practitioner treatment within the four weeks before they commit suicide -usually without the suicidal tendency being discussed [74]. Furthermore, non-psychotherapeutic medical professionals very often come into direct contact with suicidal patients: General practitioners (as “family doctors”), for example, play a role here, as they determine suicidality through their patients’ anamnestic data or they are informed of the patients’ suicidality through direct reports. In the clinical (emergency) setting, rescue workers, nursing staff or ward physicians are usually the first to come into contact with suicidal patients. After initial clinical care, they are referred to appropriate psychologists or psychiatrists for a comprehensive psychological evaluation [76].

## 9. Stigmatization of Suicidal Patients in the Health Care System

What appears to be important in stigmatization in the health care system—analogous to the conception of stigmatization according to Link and Phelan [52]—is the stereotypical view of suicidality held by the treatment providers. Depending on the “attitudes or assumptions” regarding suicidality, the therapy motivation of the affected patients is influenced [77]. By definition, the term “attitude” refers to a person’s experience-based motivation to evaluate a person, a social group or a situation. This is expressed both cognitively (through cognitions such as assumptions and beliefs) and affectively (through subjectively felt feelings and emotions) on the one hand, and through behaviours (arising from and dependent on cognitions and affects) on the other [78,79]. Accordingly, suicide-related assumptions also have a direct impact on patient contact through the behaviour of professionals towards patients [77,80]: Thus, according to Taylor et al. [80], actions with apparent high suicidal intent elicit more compassion than actions that suggest high manipulative intentions from the patient—in the sense of an appeal for help, for example, or to attract attention without actual suicidal intent. The latter could potentially result in treatment being limited to a physical examination only, for example, the mere stitching up of intentionally induced cuts. Psychotherapeutic or psychiatric treatment often takes place only partially or not at all, which has a negative impact on the mental health status of the patients [33] With regard to the aforementioned statement the authors describe the case of a 30-year-old female patient who has shown suicidal behaviour since childhood. She made numerous suicide attempts and finally stabilised successfully after changing the type of therapy. Quite a few psychotherapists had the opinion that patients of this type should no longer be sent to the ambulance service after the fourth suicide attempt and should be left to die. Today, the patient has a little daughter and lives in a happy relationship. She is happy to be alive. However, the studies on the attitudes of professionals towards suicidality—in the sense of an assessment of suicidal patients—have hardly been examined. The few studies, mostly conducted in India, show a clear stigmatization of suicidal patients by professionals [81]. A possible reason speculated here is that suicide is punishable by law in India and is accordingly perceived as a criminal offence by the general population—and thus possibly also by professionals [82]. A comparative review of international studies by Saunders et al. [83] found that clinical professionals experience more “anger”, “frustration”, “irritation” and “helplessness” towards patients who engage in suicidal behaviour than towards other patients. Self-injurious behaviour includes all arbitrary actions that intend physical injury or the failure to act in a way that prevents physical injury. Self-injurious behaviour occurs in the context of various mental disorders, such as depression, obsessive-compulsive disorder and psychotic disorders. However, self-injurious behaviour is one of the diagnostic criteria for borderline personality disorder. It is true that self-injurious behaviour can be accompanied by suicidality, but suicidality is not necessarily a component of self-injurious behaviour [73]. In the clinical setting, the majority of patients diagnosed with borderline personality disorder who display self-injurious behaviour are female. Patients often report a lack of empathy from healthcare professionals compared to other patients [83]. According to Taylor et al. [80], there is evidence that expertise on suicidality among healthcare professionals is rather insufficient to establish professional, appropriate and treatment-oriented patient contact. A Study conducted by Eilers et al. [84] investigated the differences in stigmatization of suicidality between the general population, health professionals and future health professionals still in training, also with regard to possible gender and age effects. The results show that medical professionals stigmatize significantly more than the other two groups, which show no differences in the level of stigmatization. Within the individual groups, female professionals in the age group “36–65 years” stigmatized suicide most. In this context, Norheim et al. [77] found that suicide-related attitudes could be dependent on the professional training of treatment staff. This was associated with “more positive evaluations”, which led to a less severe structural stigmatization of suicidal people by treating professionals and more expert knowledge regarding the causes of suicidality. This was shown, for example, in a decrease in agreement with the statements that suicide “cannot be justified” or that suicide “should not be talked about”. Structural stigmatization, as described above, describes how public and private institutions deal with suicidal people or those suffering from a mental disorder. In the health care system, for example, this includes inequality with somatically ill patients or the exclusion of relatives of a suicidal patient from receiving insurance benefits, as well as stigmatization by treating professionals [55]. Combines with the reported research results, the acquisition of knowledge through specialised training away from a training-independent and unaccompanied experience with suicidal patients and suicidality could thus be decisive for the development of a decrease in structural stigmatization. Thus, targeted, informative and professional training of medical and psychological staff seems to be essential for the optimal treatment of such patients.

## 10. Gender-Specific Suicidality and the Connection with (Self-) Stigmatization

In addition to the link to educational dependency on attitudinal changes towards suicide, gender also appears to be an influencing factor. For example, the study by Fox et al. [85] found that more men than women die from a suicide attempt. This finding is consistent with WHO [11] data that 1.8 times more men than women die by suicide globally each year. Suicide attempts without a lethal outcome, on the other hand, are committed mainly by young women [86]. Men, on the other hand, report fewer suicide-related thoughts and fantasies than women, while women also make more suicide plans and attempts [87,88]. This phenomenon has become known among experts as “The Gender Paradox in Suicide” [89]. 

## 11. Factors for Gender-Specific Suicidality in Connection with Gender-Specific Stigmatization

There are various approaches to explaining why such manifest gender differences in (attempted) suicide occur globally. One of the earliest approaches is based on the social construct of so-called “hegemonic masculinity” [90] This term was first introduced by Connell [91] and comes from sociological gender research. The author defines hegemonic masculinity as a social practice to secure and legitimise the dominant position of men vis à vis women or vis à vis other men perceived as “feminine” (including mentally ill men, homosexual men, transgender men, etc.) in society. In this context, women or men who are perceived as “feminine” are generally seen as “weak”, “incompetent”, etc. [92]. Empirically, it was shown that women are in fact disproportionately represented in activities that required “typically female” characteristics, such as greater “empathy”, “emotionality”, (social) “passivity” and “caring behaviour” [93,94]. This is reflected in the high proportion of female employees in the care sector, while men tend to hold leadership positions [95,96]. In this context, the so-called “sexualism”, which refers to the “gender prejudice in language”, is also noticeable [97]: Here, idioms such as “be a man” or “he fights like a man” can be found as examples of the linguistic idealisation of the male gender. Traditional male gender roles tend to be characterised by a higher attribution of qualities such as “strength”, “independence”, “risk-taking behaviour”, “individualism” (i.e., an individual’s needs are given priority over the community) and a higher socio-economic status compared to traditional female gender roles. A socially high expression of this gender role prevents men from seeking professional help for suicidal acts and depression [90,98,99]. Webster Rudmin et al. [99] examined the difference in suicide rates between the sexes in a broad study of 66 countries. The authors found four main culturally determined factors to explain this difference: “power distance” (defined as the social separation of people based on their socio-economic status), “individualism”, “uncertainty avoidance” (meaning the degree of aversion to unforeseen situations) and subjectively perceived “masculinity”. The culturally determined factor “individualism” showed a particularly strong correlation with the gender differences in suicides: Culturally individualistic countries had higher suicide rates among men than collectivistic countries (i.e.,: the common good takes precedence over the needs of the individual). However, the combination of “masculinity”, “power distance” and “uncertainty avoidance” correlated negatively with the suicide rate, meaning that a person of male gender with a high socio-economic status and a high tendency to avoid uncertainties has a lower risk of suicidal acts. In contrast, a highly pronounced social gap between rich and poor was associated with a smaller gender difference in suicide rates in the study. The results of Payne et al. [98] support this fact: they show that gender differences with regard to suicide are smaller in developing countries than in industrialized nations. The social stigmatization of women in developing countries is discussed as a possible reason for the comparatively higher suicide rates among women compared to industrialized countries, which is particularly reinforced by infertility or the birth of children out of wedlock. Finally, the study found that stabilising cultural factors have a stronger influence on the suicide rate of women than of men. Parenthood, on the other hand, generally has a protective effect against suicidal acts, although here too it is gender-specific with a stronger protective effect on women than on men [90]. To further clarify the gender paradox in suicide, a number of other factors could also be identified. For example, the death of a spouse or a divorce are risk factors for suicide for both sexes, although the effect is somewhat smaller for women. Compared to men, women often use social relationships more intensively or make use of psychotherapy to cope with their grief, which is considered a protective factor against suicidal acts [100]. Furthermore, employment status is also closely associated with both traditional gender roles and increased suicide among unemployed men. For example, Möller- Leimkühler´s [101] study showed that in times of increased unemployment, men have a higher risk of suicide than women due to the frustration of societal expectations and norms as being able to work, perform, provide for a family, etc. In addition, the choice of suicide method is discussed as a component of the gender paradox, because a gender difference could also be found here: According to this, men mostly resort to the so-called “hard suicide methods”, which are more likely to result in a lethal outcome—for example suicide by shooting, hanging or falling into the depths—while women more often choose so-called “soft suicide methods”, which guarantee a significantly higher chance of survival. These include, for example, poisoning by tablets, drugs or other substances such as cleaning agents, household chemicals, etc. [89]. The connection between stigmatization of suicidal acts and male gender becomes particularly clear in the results of a longitudinal study by Goldney, Smith, Winefield, Tiggeman & Winefield [102]: A large proportion of the male participants who stated at the first time of the study that they had been suicidal in the past year stated at the next time of the survey four years later that they had never been suicidal in their lives. A possible reason for the change in the statement is the stronger social stigmatization of suicidal men compared to suicidal women [103]. With Fox et al.’s [85] finding that suicidal thoughts are associated with a normalization/glorification of suicide and the finding that male suicidality may result in higher social stigma, there may be a fundamental gender dependency in general attitudes towards suicidality. Furthermore, current research suggests that men have more stigmatising attitudes towards suicide than women, meaning that men are more likely to see suicidal people as “weak”, “cowardly” or “stupid” [104,105]. In this way, men also devalue their own gender group and thus create a demarcation between their own group of “strong men” and the external group of “weak men” or “weak women” in order to maintain a positive self-image. As a result, the group of the “weak” is socially stigmatized. In order to avoid one’s own social assignment to this stigmatised group, therapeutic help is avoided so that the “real” assignment does not become apparent [106,107,108]. This is an implication of the “social identity” according to van Zomeren et al. [108] and the “self-categorisation theory” by Turner and Reynolds [109], according to which a person’s social identity corresponds to their identification with a group.

## 12. Age-Specific Suicidality and the Connection with Stigmatization

Age can be assumed to be another influencing factor on suicide-related attitudes: From the age of 14 on, there is a gender difference among suicides committed, which continues to increase until the age of 24 and affects the majority of the male gender. Before the age of 14, the suicide rate is so low that there is no statistical difference between the sexes [110]. Adolescents (14–17 years) and young adults (18–35 years) are more likely to commit suicide attempts than adults (36–65 years) and older people (over 66 years) [111], but they are less likely to commit completed suicide: between 0.5–1% of suicide attempts among adolescents result in a lethal outcome, while 25% of suicide attempts from adulthood onwards are fatal [112,113]. So far, there are only contradictory findings on attitudes towards suicide in the course of old age: Boldt [114], for example, examined generational differences with regard to the stigmatization of suicidal acts in a broad intrafamilial study. The author’s results showed that the younger generation was less stigmatizing towards suicide compared to the parents’ generation. Furthermore, adolescents viewed suicide as a result of societal failure rather than individual failure. Li and Philips [115] replicated these findings three decades later: the authors found that the acceptance of suicide decreases with age and suicidality is more stigmatised. However, there are also contradictory study results in this regard: The study by Batterham et al. [57] showed here that young adults would stigmatise suicide or suicidal acts more strongly than people in high adulthood—although the young adults had better knowledge regarding suicide. Pereira and Cardoso [116], on the other hand, found that adults were more stigmatizing towards suicidality compared to adolescents. This effect was particularly pronounced among the female participants in their study. Another sharp increase in the number of suicides committed by men in relation to women occurs from the age of 75. Canetto [89] speculates that this could be due to a lack of coping strategies in the face of the symptoms of old age and illness and the associated loss of everyday competence as well as severe pain. The study conducted by Eilers et al. [84] showed neither less stigmatization nor more normalization/glorification (Glorification here means the inappropriately euphemistic or romanticised portrayal of suicide) with higher age. With regard to the current debate on euthanasia, in which the deliberate causing of death in terminally ill and/or elderly people by means of medication or by stopping treatment is discussed, the connection between age and attitudes towards suicide is of correspondingly great social significance. 

## 13. Conclusions

As a conclusion from the research situation so far, no clear results can initially be derived regarding the stigmatization of suicidality in connection with age. However, it can be seen that the probability of a completed suicide increases with age. Possibly, this can also be interpreted as an indication of a tendency towards decreasing stigmatization and stronger normalisation or even glorification of suicide with higher age. 

In this context, the increase in the prevalence of dementia with increasing age should be considered when it comes to death wishes: for those over 65, the figure is around 8% and rises to over 20% for those over 80 [117]. Therefore, it must also be examined in this case to what extent a death wish is a psychopathological expression rather than a conscious intention to die before an assisted suicide is considered [118].

It is possible that the gap between theoretical content and actual practical experience with suicidality is too large and is not sufficiently compensated for by supervision, intervision and further training. Here, too, dedicated training of established or future treatment providers for suicidal people could be useful in order to enable appropriate treatment. However, it must be fundamentally questioned what goal should be achieved with special training of (future) professionals and whether a resulting lower stigmatization of suicidality could achieve the desired effect: The example of Japan clearly shows that low stigmatization is not necessarily accompanied by reduced suicide rates—the opposite is even the case there: Japan sets itself apart from other countries with very high suicide rates in an international comparison. The situation in the BeNeLux countries with liberal legislation regarding suicide and a corresponding health system should also be questioned: Here, psychiatrically and psychotherapeutically allegedly “out-treated” patients with diagnosed mental disorders as the sole illness are granted assisted suicide. In this case, there is possibly a low stigmatization of suicidality—but the therapeutic treatment mandate of suicidality is ethically doubtful if the question of suicidal patients’ capacity for judgement has not even been unanimously clarified among experts. In the Netherlands, euthanasia in the case of dementia is also permitted—even against the will of the person concerned—provided that a corresponding living will from the time before the onset of the disease is available. In February 2020, assisted suicide was also introduced as legal in Germany. This raises the question of how to deal with suicidality in countries where assisted suicide is introduced: Should training courses—e.g., along the lines of the BeNeLux countries—teach content in favour of a liberal approach to suicidality? The levels of Dutch and German suicide rates are comparably high—Belgium’s suicide rate is even considerably higher in comparison [11]. Thus, it can be concluded that a liberal approach does not have a preventive effect on those affected. A separate training should therefore aim at reducing stigmatization tendencies. This could be achieved, for example, by dispelling suicide myths such as “the suicidal person just wants to get attention” [2], creating higher empathy and with instructed change of perspective of the patient’s point of view as well as higher motivation for working with suicidal patients—after all, assisted suicide should not be considered as a real treatment alternative. This could otherwise establish another form of (self-)stigmatization: If the treating professional supports a suicide, no change of perspective would be achieved, but an affirmation of suicide as the only way out of the patient’s suffering. However, this question of how to deal with suicide has been controversially discussed since ancient times and still leads to polarising views today. A look at history shows that it has not yet been possible to establish a stigma-free approach that does not lead to extremes. For example, legal prohibitions on suicide contribute to increased stigma and increased suicide rates, as is the case in India. The romanticising-glorifying ideas of suicide described above (Japan) and also liberal legislation (BeNeLux countries) do not seem to contribute to a reduction in suicide rates. Last but not least, religious views also shape society’s approach to suicidality. Therefore, it cannot be ruled out that the health care system’s approach to mental illness has developed from religious-historical backgrounds, or that even members of the professional staff cannot completely free themselves from their own religious or philosophical attitudes beyond the learned professional opinions. For example, the long tradition of church pastoral care has proclaimed suicidality as a sin. Ultimately, this would not be the effect of professional training in treatment, but rather an archaic way of thinking that tempts practitioners to stigmatise. Consequently, a training system should constantly question its own contents and constantly investigate the effects on stigmatization tendencies as well as the consideration of assisted suicide of mentally ill people by practitioners. How such a system and corresponding educational content could look like would be another research question. At this point, however, it should be noted that the current education/training system does not seem to be sufficient to counteract the stigmatization of suicidality.

All in all, the overview shows that a lot of education to reduce stigmatization of suicide still needs to be done in the area of stigmatization towards suicidality. Ultimately, we must not lose sight of the fact that stigmatization and social exclusion of all kind cost human lives.
